# Protein Ligands in the Secretome of CD36^+^ Fibroblasts Induce Growth Suppression in a Subset of Breast Cancer Cell Lines

**DOI:** 10.3390/cancers13184521

**Published:** 2021-09-08

**Authors:** Kosar Jabbari, Garrett Winkelmaier, Cody Andersen, Paul Yaswen, David Quilici, Saori Furuta, Qingsu Cheng, Bahram Parvin

**Affiliations:** 1 Department of Biomedical Engineering, University of Nevada Reno, Reno, NV 89557, USA; kosar.jabbari@nevada.unr.edu (K.J.); gwinkelmaier@nevada.unr.edu (G.W.); codyandersen@nevada.unr.edu (C.A.); P_Yaswen@lbl.gov (P.Y.); 2 Nevada Proteomics Center, University of Nevada Reno, Reno, NV 89557, USA; quilici@unr.edu; 3 Department of Cancer Biology, University of Toledo, Toledo, OH 43614, USA; Saori.Furuta@UToledo.edu; 4 Department Cell and Molecular Biology, University of Nevada Reno, Reno, NV 89557, USA; 5 Renown Cancer Center, Reno, NV 89502, USA

**Keywords:** CD36^+^ fibroblasts, conditioned medium, breast cancer cells, 3D colonies, proteomics profiling

## Abstract

**Simple Summary:**

Human breast cancers are not fully autonomous. They are dependent on nutrients and growth-promoting signals provided by stromal cells. In order to instruct the surrounding cells to provide essential growth factors, cancer cells co-opt normal signaling molecules and mechanisms. To inhibit or potentially reverse tumor growth, our goal is to emulate this signaling and reprogram the microenvironment. For example, in a healthy mammary gland, fibroblasts (FBs) overexpress CD36; and the downregulation of CD36 is one of the hallmarks of cancer-associated FBs. Therefore, in this project, we hypothesized that signaling from CD36^+^ FBs could cause growth suppression in a subset of breast cancer cell lines. We then designed a series of experiments to validate this growth suppression and identified responsible secreted factors by the CD36^+^ FBs. These experiments suggested that three protein ligands are primarily responsible for growth suppression in a subset of breast cancer cell lines.

**Abstract:**

Reprogramming the tumor stroma is an emerging approach to circumventing the challenges of conventional cancer therapies. This strategy, however, is hampered by the lack of a specific molecular target. We previously reported that stromal fibroblasts (FBs) with high expression of CD36 could be utilized for this purpose. These studies are now expanded to identify the secreted factors responsible for tumor suppression. Methodologies included 3D colonies, fluorescent microscopy coupled with quantitative techniques, proteomics profiling, and bioinformatics analysis. The results indicated that the conditioned medium (CM) of the CD36^+^ FBs caused growth suppression via apoptosis in the triple-negative cell lines of MDA-MB-231, BT549, and Hs578T, but not in the ERBB2^+^ SKBR3. Following the proteomics and bioinformatic analysis of the CM of CD36^+^ versus CD36^−^ FBs, we determined KLF10 as one of the transcription factors responsible for growth suppression. We also identified FBLN1, SLIT3, and PENK as active ligands, where their minimum effective concentrations were determined. Finally, in MDA-MB-231, we showed that a mixture of FBLN1, SLIT3, and PENK could induce an amount of growth suppression similar to the CM of CD36^+^ FBs. In conclusion, our findings suggest that these ligands, secreted by CD36^+^ FBs, can be targeted for breast cancer treatment.

## 1. Introduction

The tumor microenvironment [1], including fibroblasts (FBs) and immune and endothelial cells, influences human breast cancer progression and malignancy [2]. Cancers are not fully autonomous but depend on growth-promoting signals provided by supporting cells, including FBs, and their biochemical and physical properties that are altered by the microenvironment [3]. Cancer-associated fibroblasts (CAFs) have emerged as potential targets for reprogramming tumor microenvironment and optimizing therapeutic strategies [4,5]. However, targeting CAFs has been challenging because of the lack of specific biochemical markers and their functional and phenotypic heterogeneity [6]. Our central hypothesis is that factors secreted from normal FBs that express CD36 could be utilized as an alternative strategy to reprogram the microenvironment and suppress the growth of certain breast tumors. CD36 is a scavenger receptor that mediates lipid uptake, is expressed in multiple cancer types [7], and accelerates tumor growth [8]. However, this receptor is downregulated in tumor-associated FBs and has been suggested as a CAF marker [9]. In contrast, in normal mammary glands, FBs express CD36 (Human Protein Atlas Database; Figure S1). Hence, understanding the normal functioning of FBs could lead to new therapies.

Our hypothesis is formulated based on two key observations: CD36 is downregulated in FBs of non-cancerous breast tissues with high mammographic density (MD) [10], where MD is associated with an increased breast cancer risk [11,12,13]; and normal FBs have been shown to have an anti-tumorigenic function through paracrine signaling [2,14,15]. Moreover, tissues with high MD, as well as tumor epithelial cells, secrete activin A, which is known to downregulate CD36 in FBs in ex vivo samples [10] as well as 3D models of MD [16]. Activin A is one of the secreted macromolecules that is overexpressed by cancer cells [17,18]; it is a member of the TGFβ superfamily of cytokines and, like TGFβ, signals through pSmad 2/3 effectors to activate cell cycle checkpoints in normal cells. Hence, by identifying factors secreted by CD36^+^ FBs, an improved therapeutic approach may be elucidated for breast cancer therapy.

Motivated by the above insights, in earlier work [19], we showed that CD36 in normal primary mammary FBs is downregulated as a result of exposure to activin A; and overexpression of CD36 in FBs in cocultures with 3D colonies, either inhibits colony growth or reverses aberrant basal and lateral polarities, as a function of the breast cancer subtypes. In this study, we further investigate how the signaling from the CD36^+^ FBs induces growth suppression for reprogramming the microenvironment. Our investigations encompass the interplay between activin A and CD36 expression in normal primary FBs; growth suppression as a result of exposure of the tumor cells to the CM of CD36^+^ FBs in multiple cell lines and a partial mechanism via identifying relevant transcription factors, and identifying active factors and their effective concentrations that induce growth suppression. 

## 2. Materials and Methods

### 2.1. Cell Culture and Maintenance 

Primary human breast fibroblast (FBs) (Zenbio, Durham, NC, USA, FM1), human triple-negative breast cancer cell lines MDA-MB-231 (ATCC, Manassas, VA, USA, HTB26), Hs578T (ATCC, HTB126) and BT-549 (HTB122), human ERBB2^+^ breast cancer cell line SKBR-3 (ATCC HTB30), and human non-malignant breast cell line MCF10A (ATCC, CRL-10317) were tested for mycoplasma contamination using an ATCC 30-1012K kit. Cells were maintained at 37 °C in a humidified incubator containing 5–7.5% CO_2_. FBs were cultured in DMEM (ThermoFisher, Bethesda MA, USA, Technology, 11965) supplemented with 10% fetal bovine serum (FBS, ThermoFisher 10438034) and 1% Pen/Strep (ThermoFisher, 15140122). MDA-MB-231 was cultured in DMEM supplemented with 10% FBS. Hs578T was cultured in DMEM with 10% FBS and 0.01 mg/mL of insulin (Sigma Aldrich, St. Louis, MO, I2643). SKBR-3 was cultured in RPMI (ATCC 30-2001) supplemented with 10% FBS. BT-549 was cultured in RPMI supplemented with 10% FBS and 0.023 U/mL insulin (e.g., active unit) (Sigma Aldrich, I2643). MCF10A cells were cultured in DMEM/F12 medium (ThermoFisher, 11320-033) supplemented with 5% horse serum (Cytiva, Marlborough, MA, USA, ShH30396.03HI), 10 ng/mL epidermal growth factor (ThermoFisher, 11320-033), 50 ng/mL cholera toxin (Sigma Aldrich C8252), 200 µg/mL insulin (Sigma Aldrich I2643), 100 ng/mL hydrocortisone (Sigma Aldrich H0888), and 1% Pen/Strep (ThermoFisher, 15140122).

### 2.2. 3D Cultures of Epithelial Cells

Epithelial cells were cultured in a 48-well plate (3–4 repeats) or 96-well plate (3–6 repeats) using the 3D on-top method [20]. Briefly, a thin layer of approximately 200 µL or the equivalent of (17–20 µL/cm^2^) of Matrigel (Corning 356243) was spread evenly on the surface of a pre-chilled plate and incubated at 37 °C for 10 min to gel. Cells were suspended in the culture medium containing 5% Matrigel at a seeding density of 10,000 cells/cm^2^ for TNBC lines, 15,000 cells/cm^2^ for HER2^+^ line, and 20,000 cells/cm^2^ for the non-malignant cell line unless otherwise specified. Cell suspensions were added to each well on the base Matrigel layer and incubated in a 37 °C humidified chamber for 24 h. The next day (considered as day 0), the culture medium was replaced by the treatment medium (described later in detail for each experiment) and thenceforth replenished with a fresh treatment medium after 48 h. On day 4 of the experiments, unless otherwise specified, the plates were washed with PBS three times and fixed with 10% formalin (or 4% fresh PFA), followed by DAPI staining and quantitative analysis.

### 2.3. CD36 Transfection of Fibroblasts (FBs) and Validation

Fibroblasts (FBs) were transfected for CD36 overexpression and knockdown using CRISPR-cas9 technology from Santa Cruz Biotechnology (SC400233-ACT/KO2), per previously published method [19]. The guide RNA for activation is 5′-AGCATCTGAGAATATGTTAG-3′, and the guide RNAs for knockout are 5′-CTCACTCACCTGTACGTATA-3′, 5′-ACCTTTATATGTGTCGATTA-3′, and TGGGCTGTGACCGGAACTG-3′. The altered CD36 expression levels were validated using fluorescence microscopy, coupled with quantitative image analysis methods, outlined later. Briefly, cells were seeded into 12-well tissue culture plates at a density of 10,000 cells per well and were incubated in 2 mL of antibiotic-free standard growth medium for 24 h. Proliferating cells, at 60–80% confluency, were then transfected with CRISPR Activation/Knockdown Plasmid, as described by the manufacturer. Solutions containing Plasmids or Transfection Reagent were mixed with the Transfection Medium at defined volumes to make the CD36 Activation/Knockdown Transfection Complex. After a 20-min incubation, the antibiotic-free growth medium of the cells was refreshed, and 113 µL of CD36 Activation/Knockdown Transfection Complex was added dropwise to each well; the medium was gently mixed by swirling the plates, and cells were then incubated for at least 24 h under normal culture conditions. Next, the medium containing the Transfection Complex was removed from the cells, and cells were cultured in normal conditions for at least three weeks to recover and confluence. Negative control cells were transfected with empty plasmid vectors. The recovered cells were stained with an anti-CD36 antibody (Santa Cruz Technology SC73643 or Novus NB400-144) for validation. CD36 expression was validated for each new batch of transfection and after every passaging.

Immunofluorescence microscopy analysis of transfected cells indicated that the transfection efficiency was around 75%, and the population was stable for at least four passages. In addition, proliferation rates of CD36^+^ and CD36^−^ fibroblasts did not differ significantly.

### 2.4. CD36 Expression in Normal Primary Fibroblasts under the Influence of Activin A

Normal mammary primary FBs were exposed to 2.25 or 20 ng/mL concentrations of recombinant human Activin A (ThermoFisher, PHG9014) for 3–5 days. The culture medium was replenished every two days with activin A. Samples were fixed and imaged with wide-field microscopy. The CD36 expression was quantified per field of view per cell.

### 2.5. Treatment of the Tumor Cells with the CM of CD36^+^ FBs

#### 2.5.1. Collecting the Conditioned Medium from FBs

Transfected normal primary FBs, CD36^+^ or CD36^−^, were seeded at 50% confluency in T-75 tissue culture flasks and incubated for at least 3 days to establish a stable cell population. Samples were washed with serum- and phenol red-free DMEM (Gibco, 31053) 2–3 times and incubated in the same media in the humidified incubator. After 48 h, the CM from CD36^+^ and CD36^−^ were separately collected and centrifuged at 2000× *g* for 10 min to remove the large floating residues of possibly dead cells; the supernatant was filtered through a 0.2 μm syringe filter if needed. Then, the CM was concentrated using 3 kDa centrifugal filter units (Amicon UFC8003) at a defined speed and time per established protocol from the manufacturer. From the concentrated CM, the portion corresponding to the identified number of fibroblasts was used. An equivalent of 1:10 ratio of epithelial cells to fibroblasts was added to the growth medium of each condition in the experiments.

#### 2.5.2. Treatments of 3D-Cultured Epithelial Cells

3D-cultures of MDA-MB-231, BT-549, and SKBR-3 cells were treated for 4, 4, and 6 days, respectively. Untreated cells were also used as control. The CM, augmented with the secretome of FBs, was replenished every other day.

#### 2.5.3. Heat Inactivation Assay

MDA-MB-231 cells, seeded at a density of 5000 cells/cm^2^ in 3D culture, were treated with heat-inactivated CM of CD36^+^ FBs. Three experimental conditions were investigated by boiling the CM, followed by adding RNase or DNase. First, the conditioned medium, collected from CD36^+^ FBs, was boiled at 95 °C for 10 min to denature the large proteins and to break up exosomes to release their contents, and then centrifuged at 5000× *g* for 10 min at 4 °C. Second, either RNase A (Thermo Scientific EN0531) was applied at a concentration of 100 µg/mL and incubated at 37 °C for 30 min, or DNase (QIAGEN 1010395) was applied at a concentration of 100 µg/mL (containing 5 mM Mg^2+^) and incubated in room temperature (RT) for 20 min. Next, to completely inactivate the DNAs and RNAs, all solutions were transferred separately to glass tubes and boiled again, followed by centrifugation at 5000× *g* for 10 min at 4 °C. Finally, for each condition, treated CM was supplemented with 10% FBS, added to corresponding wells in the 48-well plate on 3D cultured MDA-MB-231 cells, and replenished every two days for a total of 7 days.

#### 2.5.4. Protein Fractionation of the CM of CD36^+^ FBs

The growth properties of the MDA-MB-231 cells, seeded at a density of 5000 cells/cm^2^ in 3D culture, were used to identify to which protein fractions contribute to growth inhibition. Untreated cells were used as the negative control, and cells treated with CM from CD36^+^ FB were used as the positive control. The CM collected from CD36^+^ fibroblasts was filtered using 50 kDa (Amicon UFC505024) filter units at 14,000× *g* for 10 min at RT, and the CM containing proteins larger than 50 kDa was collected. The flow-through containing the proteins smaller than 50 kDa was filtered with 3 kDa filter units (Amicon UFC500396), and the medium containing proteins between 3–50 kDa was collected. The fractioned CM was supplemented with FBS as needed, added to corresponding wells in the 48-well plate on 3D cultured MDA-MB-231 cells, and replenished every two days for a total of 7 days.

#### 2.5.5. Analysis of Factors Associated with Exosomes

Secretion of exosomes by CD36^+^ FBs was reduced or increased by adding with 1 μM tipifarnib (low exosome) or 10 μM sitafloxacin (high exosome), respectively, for 72 h per published protocol in serum-free media [21]. Conditioned medium from untreated CD36^+^ FBs was used as the control. Next, the conditioned media containing low or high exosomes were collected and added to the 3D-cultured MDA-MB-231 and incubated for five days. The CM was replenished once.

### 2.6. Apoptosis Assay

3D-cultured MDA-MB-231 cells were used to investigate the role of apoptosis in growth inhibition. Positive and negative controls included apoptosis inducer Staurosporine (STS) from Abcam (ab120056) at 5 μM concentration, and pan-caspase inhibitor (Z-VAD-fmk from AdooQ Bioscience 187389-52-2) at a concentration of 20 μM. The experimental conditions included CD36^+^ CM with or without pan-caspase inhibitor. Another control was DMSO (as an empty vehicle), which is the solvent for the pan-caspase inhibitor. The culture continued for 24 h until apoptosis in the positive control was observed. At this time, samples were first stained with propodium iodide (PI) and Hoescht and imaged. The same samples were washed and fixed with 10% Formalin and stained for cleaved caspase-3 (Abcam, ab32042) and DAPI.

### 2.7. Proteomics Profiling

225 µg of proteins were collected from the CM of four million cells from CD36^+^ or CD36^−^ FBs. For each condition, three replicates, each containing 50 µg, were constructed. The rest were used to build the library. Subsequently, the UNR proteomics facility utilized the following protocol.

#### 2.7.1. Protein Digestion 

Isolated protein samples were digested and desalted in triplicate according to protocols in the published literature [22]. Protein concentration was determined using a bicinchoninic acid assay (BCA) (Thermo-Fisher Scientific, San Jose, CA, USA). Proteins were reduced and alkylated and subjected to methanol-chloroform precipitation prior to digestion with endoproteinase Lys-C (Wako, Richmond, VA, USA), followed by digestion with trypsin (Promega, Madison, WI, USA), desalting, and concentration using C_18_ Sep-Pak cartridges (Waters).

#### 2.7.2. Peptide Quantification 

Peptides were mass tagged using Thermo-Fisher’s TMT 10-plex isobaric label kit (Cat # 90061) following the included protocol. Labeled samples were pooled for analysis.

#### 2.7.3. Basic Reversed-Phase Fractionation 

The pooled TMT-labeled peptides were fractionated by basic pH reversed-phase (BPRP) fractionation on an Ultimate 3000 HPLC (Thermo Scientific) using an integrated fraction collector. Elution was performed using a 10 min gradient of 0–20% solvent B followed by a 50 min gradient of solvent B from 20–45% (Solvent A 5.0% acetonitrile, 10 mM ammonium bicarbonate pH 8.0, Solvent B 90.0% acetonitrile, 10 mM ammonium bicarbonate pH 8.0) on a Zorbax 300Extend-C18 column (Agilent) at a flow rate of 0.4 mL/min. A total of 24 fractions were collected at 37 s intervals in a looping fashion for 60 min, then combined to produce a total of 12 fractions. Peptide elution was monitored at a wavelength of 220 nm using a Dionex Ultimate 3000 variable wavelength detector (Thermo Scientific). Each fraction was then centrifuged to near-dryness and desalted using C_18_ Sep-Pak Cartridges, followed again by centrifugation to near-dryness and reconstitution with 20 uL of 5% acetonitrile and 0.1% formic acid.

#### 2.7.4. Liquid Chromatography and Mass Spectrometry 

This step processed each fraction to identify and quantify the sample. Peptide fractions were separated using an UltiMate 3000 RSLCnano system (Thermo Scientific, San Jose, CA, USA) on a self-packed UChrom C18 column (100 um × 35 cm). Elution was performed using a 180-min gradient of solvent B from 2–27% (Solvent A 0.1% formic acid, Solvent B acetonitrile, 0.1% formic acid) at 50 °C using a digital Pico View nanospray source (New Objectives, Woburn, MA, USA) modified with a custom-built column heater and an ABIRD background suppressor (ESI Source Solutions, Woburn, MA, USA). Briefly, the self-packed column tapered tip was pulled with a laser micropipette puller P-2000 (Sutter Instrument Co, Novato, CA, USA) to an approximate id of 10 µm. The columns were then packed with 1–2 cm of 5 µm Magic C18 followed by 35 cm of 1.8 µm UChrom C18 (120A) at 9000 psi using a nano-LC column packing kit (nanoLCMS, Gold River, CA). The mass spectral analysis was performed using an Orbitrap Fusion mass spectrometer (Thermo Scientific, San Jose, CA). TMT analysis was performed using an MS3 multi-notch approach [23]. The MS1 precursor selection range was 400–1400 m/z at a resolution of 120 K and automatic gain control (AGC) target of 2.0 × 10^5^ with a maximum injection time of 100 ms. Quadrupole isolation at 0.7 Th for MS^2^ analysis using CID fragmentation was performed in the linear ion trap with a collision energy of 35%. The AGC was set to 4.0 × 10^3^ with a maximum injection time of 150 ms. The instrument was operated in a top-speed data-dependent mode with a most intense precursor priority with dynamic exclusion set to an exclusion duration of 60 s with a 10 ppm tolerance. MS2 fragment ions were captured in the MS3 precursor population. These MS3 precursors were isolated within a 2.5 Da window and subjected to high energy collision-induced dissociation (HCD) with a collision energy of 55%. The ions were then detected in the Orbitrap at a resolution of 60,000 with an AGC of 5.0 × 10^4^ and a maximum injection time of 150 ms. The data were extracted using a Proteome Discoverer (Thermo Scientific, San Jose, CA, USA). All samples were analyzed using Sequest (Thermo Fisher Scientific, San Jose, CA, USA), version v.27, rev. 11. Scaffold PTM and Scaffold Q+ (version Scaffold_4.2.1, Proteome Software Inc., Portland, OR, USA) were used to validate the site of phosphorylation and quantified using the TMT reporter ions.

#### 2.7.5. Signal Normalization 

Normalization factors were computed per sample as the sum of the raw protein values (across all proteins) divided by the maximum pool sum, following the TMT Thermo-Fisher protocol and manual. Principal component analysis (PCA) was applied to experimental conditions, performed in triplicate, to assess and remove any outliers. Raw values were normalized by dividing by the sample-specific normalization factor. Normalized data were log2-transformed, and ANOVA was performed followed by a Tukey posthoc test on any proteins with a statistically significant change (adjusted *p*-value < 0.05). Bioinformatics analysis was performed using Scaffold DIA (Protomesoftware).

### 2.8. Human Recombinant Proteins

Human recombinant proteins, secreted by HEK293 cells, were commercially acquired for SLIT3 (Novus Biological, 9255-SL-050), FBLN1 (Novus Biological, 9007-FB-050), PENK (Novus Biological, H00005179-P01-2ug), and TIMP2 (Novus Biological, NBP2-61356). To validate their growth inhibitory effects, neutralizing antibodies for SLIT3 (Novus Biological, AF3629-SP), FBLN1(Novus Biological, NBP1-84725-25ul), and PENK (Novus Biological, NBP1-90944-25ul), at a concentration of 1 µg/mL, were added to the CD36^+^ CM and applied to 3D-cultured MDA-MB-231 cells for 4 days. In all experiments, cells were fixed and stained with DAPI. The positive control was the CM of CD36^+^ FBs, which is estimated to have 1.5 µg/well of proteins.

### 2.9. Inhibition of KLF10 Transcription Factor

CD36^+^ CM with or without 12 μg/mL of KLF10 inhibitor (Sigma #533976) was added to 3D-cultured MDA-MB-231 cells for 4 days. The toxicity of the KLF10 inhibitor and its solvent (DMSO) was evaluated by adding it to the growth medium.

### 2.10. Immunofluorescence Staining

For detecting live and dead cells, a culture medium containing 10 µg/mL of propidium iodide (PI) (Alfa Aesar, Haverhill, MA, J66764MC) and Hoechst (BD Biosciences, Franklin Lakes, NJ, 561908) was added directly to cell cultures prior to fixation, followed by fluorescent microscopy.

For CD36, YY1, Ki67, Cyclin E1, and KLF10 staining, cell cultures were washed three times with PBS (with Ca^2+^ and Mg^2+^, ThermoFisher14040-133) and fixed at room temperature in 4% PFA (ThermoFisher, 28908) for 15 min. After three PBS washes, cells were permeabilized using a Triton X-100 solution (Sigma Aldrich, T8787) for 10 min and then incubated for 1 h in a blocking solution containing bovine serum albumin (BSA, Sigma Aldrich, A7638) in PBS on a shaker at RT. The primary antibody was diluted in the blocking solution and applied to cells overnight at 4 °C. The following day, samples were washed three times in PBS (15 min per wash). Secondary antibody was diluted in blocking solution applied to samples for 1 h. Cells were washed three times in PBS (15 min per wash). Finally, the nuclei were counterstained with 250 ng/mL 4′-6-diamidino-2-phenylindole (DAPI, ThermoFisher, D1306). For cleaved caspase-3 staining, the same procedure was applied, except that 1% BSA containing 0.06% Triton X-100 was used as the washing buffer instead of PBS. Specific conditions and concentrations for each target are indicated in Table 1.

### 2.11. Fluorescence Microscopy

The readout for each molecular endpoint is based on fluorescence microscopy, where our lab has excelled in the development of quantitative assays [24,25]. Typically, 60 to 300 cells are present per field of view with a 10× objective, which provides significant power for data analysis. On average, two to five fields of view are imaged per well, and there are three to six wells sampled per condition. Samples were imaged with an EVOS FL Auto Imaging System equipped with an AMEP 4633 10× phase objective (0.25 of NA and 6.9 mm of WD) and a 40× objective (0.8 ND and 3.3 mm working distance). The excitation lasers were set at 385, 488, and 568 nm for DAPI, Alexa 488, and 568 fluorophores, respectively. All other imaging parameters were kept constant for all specimens.

### 2.12. Quantification of Molecular Endpoints

For quantitative analysis of CD36, YY1, Ki67, KLF10, propidium iodide, and cleaved caspase-3, samples were DAPI stained to provide a context for each molecular endpoint. All images were background corrected against an empty well for each excitation frequency. Quantitative analysis of each molecular endpoint utilizes robust image analysis that has been developed in our laboratory by first segmenting each nucleus in the image [26].

To quantify the CD36 expression in fibroblasts, nine fields of view were captured per condition with a 10× objective. Images were binarized with the same threshold. The total fluorescent signal per field of view was averaged against the number of nuclei in the same field.

To quantify the nuclear-bound YY1 transcription factor, 10 fields of view per condition were randomly selected and imaged with a 10× objective. Colonies were segmented from the background with a constant threshold on the DAPI stained channel. Subsequently, the average YY1 expression was computed in each colony. Having quantified the average YY1 expression per colony, consensus clustering [27] was applied to conclude that there were two populations of low and high-YY1 expressing colonies for all conditions. Finally, the number of colonies in each population, per condition, was computed.

To quantify the Ki67 expression, 30 fields of view per condition were imaged with a 10X objective, and the average Ki67 signal per nucleus per field of view was computed. The two populations were identified by consensus clustering [27], and the number of cells in each population per condition, was also computed.

One of the reasons for using consensus clustering is automatic threshold selection for identifying different populations that can be statistically validated.

To quantify the frequency of the cleaved caspase-3 positive cells per condition, the mean fluorescent signals for positive (e.g., STS treatment) and negative controls (e.g., untreated, inhibitor) were computed, a threshold was set in the middle and applied to all conditions. Next, the frequencies of positive cleaved caspase-3 were quantified and normalized to the total number of cells per condition.

To quantify the KLF10 and Cyclin E1 expression, 12 to 20 images per condition were segmented using the DAPI channel, and the total fluorescent signal per field of view was averaged against the number of nuclei in the same field. KLF10 was measured within the cell area, and Cyclin E1 was measured within each nucleus.

### 2.13. Visualization of Images

All images in a study (e.g., positive and negative controls, treatment) were enhanced on the same linear scale between min and max intensities. However, all quantitative analyses were performed with raw data. In some cases, a 5× or 40× objective was used for visualization.

### 2.14. Figures and Statistical Analysis

Results are shown in the scatter bar chart, where each point, in a bar chart, represents either the total number of cells per field of view or the average fluorescent per cell per field view. The error bar corresponds to the standard error of the mean for all fields of view and replicates per condition. Differences between groups were identified using the student’s *t*-test, and their significance is displayed with either one or two asterisks.

## 3. Results

### 3.1. CD36 Expression in FBs Is Reversible and Anti-Correlated with the Exposure to Activin A

In a previous work [19], we showed that activin A secreted from tumor epithelial cells decreases CD36 expression in FBs in a coculture assay and that CD36 expression was inversely correlated to the concentration of activin A in a dose-dependent manner. In the present study, primary FBs were treated with a nominal concentration of activin A [28] in tumors, at a minimal concentration of 2.25 ng/mL and as high as 20 ng/mL, and the CD36 expression in FBs was quantified under three experimental conditions: (i) three days of treatment followed by fixation, (ii) five days of treatment followed by fixation, and (iii) three days of treatment followed by replacing activin A-containing medium with the normal culture medium, followed by two days of incubation before fixation. In addition, the CD36 expressions for transfected and normal FBs were used as controls. We found that activin A-induced downregulation of CD36 expression is reversible once activin A is removed from the medium (Figure S2). Reversion of the CD36 in FBs is one of the requirements for reprogramming the microenvironment.

### 3.2. Growth Suppression by the CM of CD36^+^ FBs Is Subtype Dependent

The number of cells or colonies was used as an endpoint to quantify growth suppression as a result of exposure to the CM of CD36^+^ FB for each cell line.

#### 3.2.1. The Triple-Negative Breast Cancer (TNBC) Cell Lines MDA-MB-231, BT-549, and Hs578T Are Sensitive to the CM of CD36^+^ FBs

**Figure 1:**
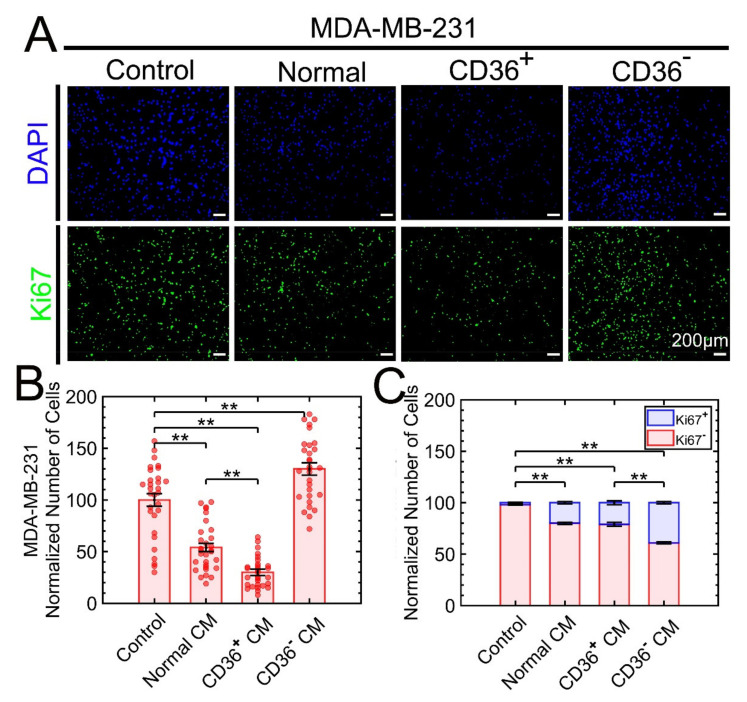
The 3D growth of MDA-MB-231 is suppressed as a result of exposure to the CM from CD36^+^ FBs. (**A**) The number of cells (DAPI) and their proliferation status (Ki67) is monitored and visualized by fluorescent microscopy under control and exposure to the CM from normal, CD36^+^, or CD36^−^ FBs. (**B**) The number of cells was quantified for each condition based on the nuclear counterstain. (**C**) Frequency of low and high expressing Ki67 cells was quantified for each condition. For (**B**,**C**), quantitative data were normalized to control (untreated cells). ** *p* < 0.001.

MDA-MB-231, originating from a patient with adenocarcinoma pathology, represents a mesenchymal phenotype and grows along a two-dimensional plane on Matrigel [24]. Previously, we showed that coculture of CD36^+^ FBs with MDA-MB-231 induced growth suppression in the absence of direct epithelial-fibroblast contact in Boyden chambers [19]. Here, 3D colonies of MDA-MB-231 cells were exposed to the CM of primary normal, CD36^+^, or CD36^−^ FBs. After four days of incubation, the samples were fixed, stained with DAPI and Ki67, and the number of cells was counted using automated image analysis. When the tumor cells were exposed to the CM from CD36^+^ or CD36^−^ FBs, there was a significant growth suppression or promotion, respectively (Figure 1), in terms of the number of cells. In addition, proliferation analysis of MDA-MB-231 for all treatment conditions revealed two subpopulations of low and high Ki67 expressing cells. Compared to the CD36^+^ CM, exposure to the CM from CD36^−^ produced a larger population of high-expressing Ki67 levels. It is feasible that there are two subpopulations of MDA-MB-231, with one being more resistant to the CM of CD36^+^ FBs. Besides, there is evidence for two subpopulations of MDA-MB-231, with one being hyaluronan (HA) receptor-positive with higher local metastasis [29].

**Figure 2:**
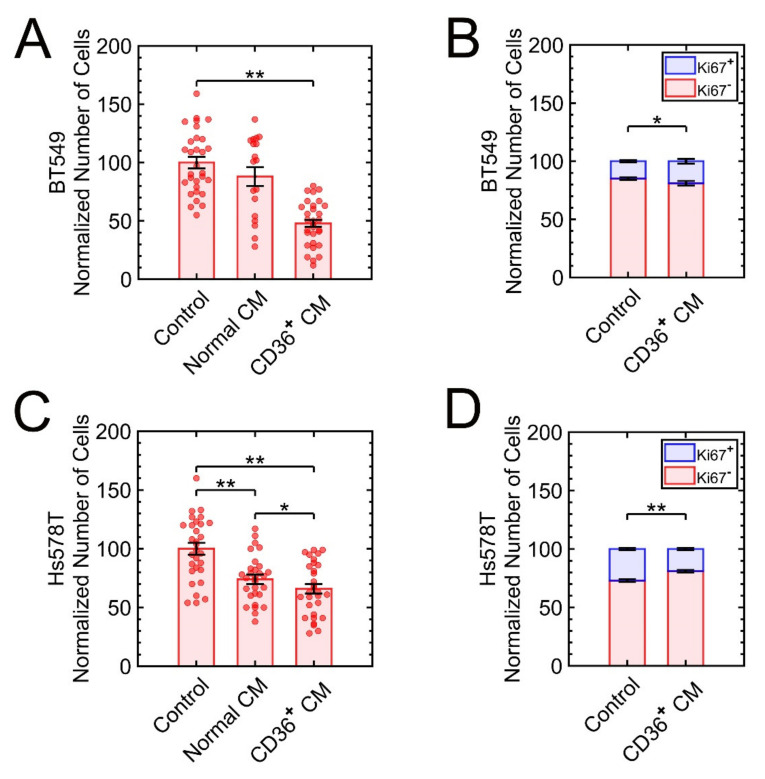
The 3D growths of BT-549 and Hs578T are suppressed as a result of exposure to the CM from CD36^+^ FBs. (**A**,**B**) The numbers of cells for BT549 and the frequencies of high versus low expressing Ki67 cells are quantified. (**C**,**D**) The numbers of cells for Hs578T and the frequencies of high versus low expressing Ki67 cells are quantified. Quantitative data were normalized to control (untreated cells). * *p* < 0.05, ** *p* < 0.001.

BT-549 and Hs578T are TNBC with infiltrating ductal carcinoma and carcinoma sarcoma features, respectively. Both grow as stellate colonies [30,31]. These cell lines were cultured and exposed, separately, to the CM of CD36^+^ or CD36^−^ fibroblasts for four days. Subsequently, the number of cells was quantified using automated image analysis (Figure 2), with the results indicating growth suppression in both cell lines.

#### 3.2.2. ERBB2+ Cell Line of SKBR3 Is Resistant to the CM of CD36^+^ FBs

SKBR3 cells, known to form three-dimensional “grape-like” colonies, were exposed to the CM of FBs, as before. After six days of treatment, the colonies were fixed, and no significant growth suppression was observed. Previously, we showed that the YY1 transcription factor plays a role in reversing aberrant polarity [19] and has a causal effect on the regulation of ERBB2 [16]. YY1 is involved in differentiation and proliferation, is overexpressed in cancer [32], and its genetic manipulation has been associated with phenotypic reversion between MCF10A and MCF7 [33]. Thus, we opted to investigate the regulation of YY1 as a result of exposure to the CM of CD36^+^ FB for this cell line. Consensus clustering identified two subpopulations of YY1-expressing colonies, and further analysis quantified the frequencies of low- and high-YY1-expressing colonies in each condition. Although the frequency of high-YY1-expressing colonies was significantly reduced after exposure to the CM of CD36^+^ FBs (Figure S3), downregulation of YY1 had no impact on the endpoint (e.g., number of colonies, size of colonies). Resistance to the CM of the CD36^+^ FBs may be due to the non-reversible expression of ERBB2 in SKBR3 that lead to the loss of proliferative suppression.

### 3.3. MDA-MB-231 Is Used to Investigate Properties of Growth Suppression as a Function of Exposure to the CM of CD36^+^ FBs

We selected the sensitive cell line of MDA-MB-231, cultured on Matrigel, for a more in-depth study that includes investigating growth suppression as a result of acute versus continuous exposure and apoptosis versus growth arrest. We also examined whether growth suppression is induced by nucleic acid sequences or proteins and the involvement of exosomes.

#### 3.3.1. Acute or Continuous Exposures to the CM of CD36^+^ FB Induce Growth Suppression

To investigate the difference in growth suppression, in cancer cells, between continuous and acute exposures to the CM of CD36^+^ FBs, we designed an experiment with three conditions: (i) control, (ii) acute exposure for two days, and (iii) continuous exposure with three replenishments over a seven-day period. Both acute and continuous exposure induced growth suppression, with the continuous exposure having an increased suppression response (Figure S4).

#### 3.3.2. Growth Suppression Is Primarily due to Apoptosis

**Figure 3:**
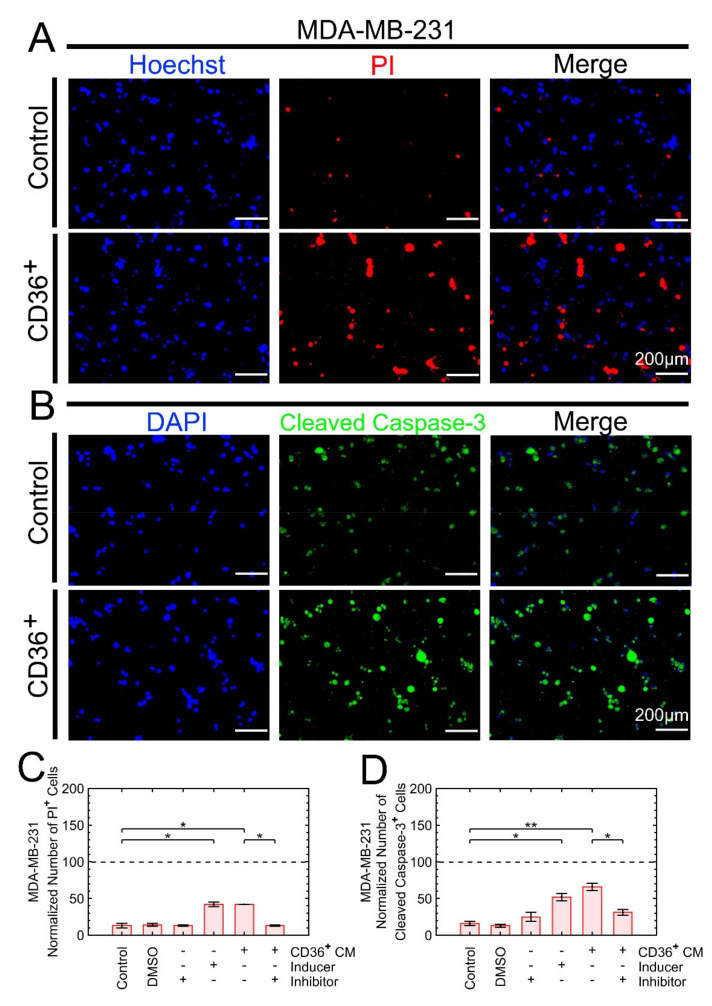
Exposure to the CM of CD36^+^ FBs increases the frequency of apoptotic cells. (**A**,**C**) PI staining indicated a higher frequency of cell death with exposure to the CM of CD36^+^ FBs. (**B**) Cleaved caspase-3 staining was monitored with fluorescent microscopy. (**C**,**D**) Inducer and inhibitor of apoptosis pathway enabled the selection of a threshold for quantitative analysis. In both cases, the frequencies of apoptotic cells, as the result of exposure to the CM of CD36^+^ FB, were the same as the treatment with apoptosis inducer. * *p* < 0.05, ** *p* < 0.001.

Phase image microscopy of the MDA-MB-231 cell exposed to the CM of CD36^+^ FB indicated potential apoptotic signatures because of the shrinkage of the cell membrane and/or fragmentation of cellular components. In addition, the number of cells at the end of the culture was always less than the initial seeding density. Hence, MDA-MB-231 was stained on day two with Hoechst and PI (stains cells with damaged membrane) and cleaved caspase-3 antibody to verify dell death. The rationale for selecting day 2 is to avoid cells be detached and washed. The control cells were exposed to staurosporine as an apoptosis inducer, a pan-caspase inhibitor, and DMSO. These experiments revealed a higher frequency of cell death and cleaved caspase-3 expressing cells when samples were exposed to the CM of CD36^+^ FBs (Figure 3). Conversely, the addition of pan-caspase inhibitor to the CM of CD36^+^ FBs reduced the frequency of apoptotic cells by approximately 30%.

In addition, staining for cyclin E1 did not indicate G1/S arrest in samples exposed to the CM of CD36^+^ FBs (Figure S5). This is potentially due to earlier results of Section 3.2.1, where we showed that there is a sensitive and resistive subpopulation of the MDA-MB-231 cell line. While the sensitive subpopulation goes through apoptosis and, detached and washed out on day 2, the resistive subpopulation continues to proliferate. Hence, the resistive subpopulation continues to express cyclin E1.

#### 3.3.3. Growth Suppression Is Likely Induced by Proteins

**Figure 4:**
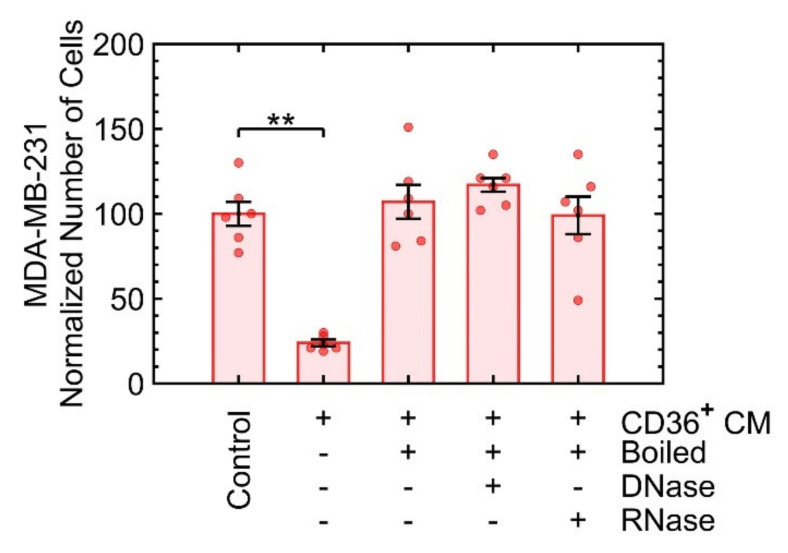
The heat inactivation assay coupled with the enzymatic treatment of the CM of CD36^+^ FBs indicates that proteins are likely to be involved in growth suppression. The cell count, on day 7, was quantified as a result of exposure to the boiled CM (i) by itself (third bar from right), (ii) with DNase, and (iii) with RNase. ** *p* < 0.001.

A heat inactivation assay was used to determine whether nucleic acid sequences or protein complexes in the CM of CD36^+^ FBs were responsible for growth suppression. The heat inactivation assay denatures large proteins, degrades RNA, and breaks up lipid membranes of the vesicles to release their contents. Subsequently, RNase or DNase enzymes were added to the heated CM to remove any remaining RNA or DNA compounds. The number of MDA-MB-231 cells, after seven days, was used as an endpoint for each treatment of the CM of CD36^+^ FBs. Because the heat inactivation assay and the addition of DNase or RNase interfered with the growth suppression (Figure 4), proteins are likely candidates.

**Figure 5:**
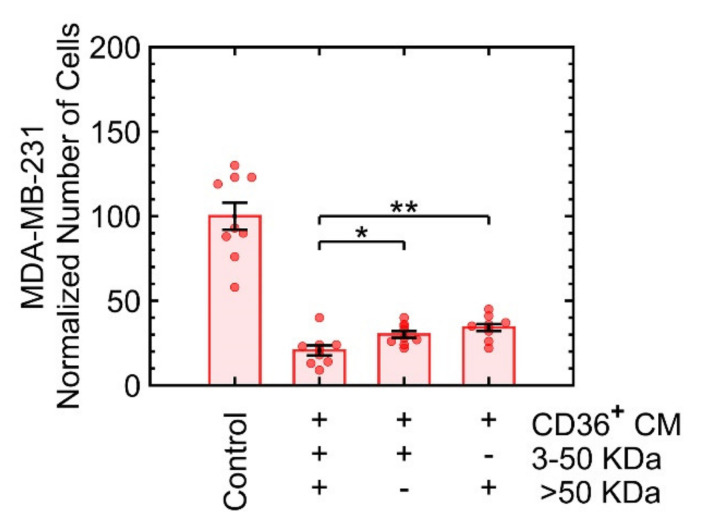
Proteins in the range 3–50 KDa or larger than 50 KDa induce growth suppression in MDA-MB-231. * *p* < 0.05, ** *p* < 0.001.

A follow-up experiment was performed to fractionate protein complexes based on their size and quantify the effect of each fraction on the growth of MDA-MB-231. Fractionation included ranges of 3 to 50 kDa and larger than 50 kDa. Both fractions induced growth suppression independently (Figure 5). The selection of the filter size was a compromise between the known range of cytokines [34] and the commercial availability of the filters.

#### 3.3.4. Exosomes Are Not the Predominant Factors in Growth Suppression

**Figure 6:**
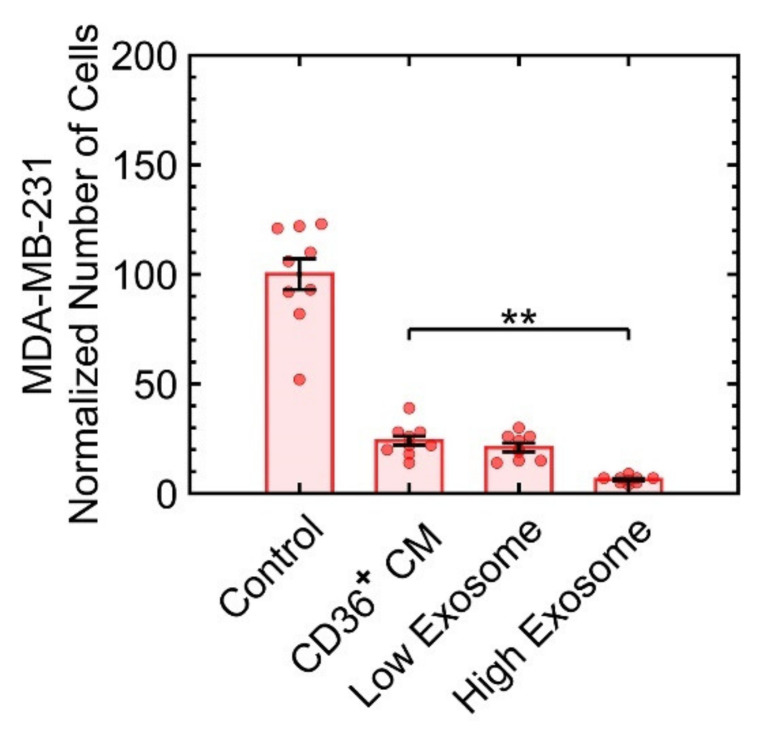
Induced high biogenesis of exosomes marginally increases growth suppression. At low exosome production, there was no statistical significance in growth suppression compared to the treatment with the CM of CD36^+^ FB. ** *p* < 0.001.

In physiological conditions, signaling proteins can be soluble or encapsulated in exosomes and microvesicles. Since the biogenesis of the exosomes can be manipulated [21], we investigated their impact on growth suppression. The culture condition of the CD36^+^ FB was supplemented with 1 μM tipifarnib or 10 μM sitafloxacin for low and high exosome production, respectively [21]. For each condition of low or high exosome biogenesis, the CM was collected, added to the culture condition of MDA-MB-231, incubated, and samples were then fixed, stained, and imaged with fluorescent microscopy to count the number of cells. The results indicated that only at high biogenesis of exosomes, a slight increase in growth suppression was observed (Figure 6). In other words, protein ligands or microvesicles appear to be the major factors in growth suppression.

### 3.4. Secretomes of CD36^+^ versus CD36^−^ FBs Reveal Differentially Expressed Proteins

**Figure 7:**
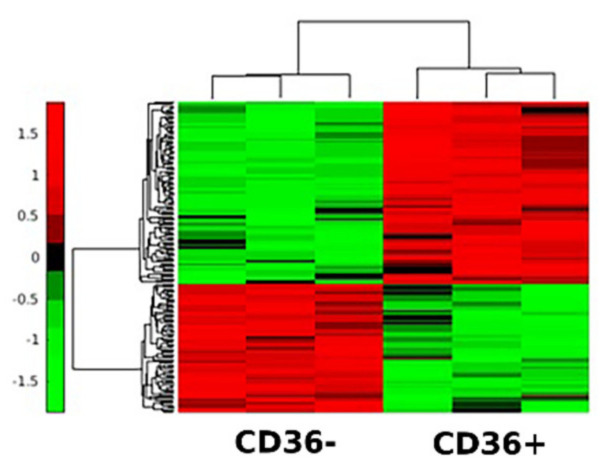
Differential profiling between the CM of CD36^+^ and CD36^−^ FBs identifies 187 proteins with *p*-values of less than 0.05.

To identify proteins responsible for growth suppression, we profiled the CM of CD36^+^ and CD36^−^ FBs. For each condition, 225 µg of total protein was collected. Three technical replicates were carefully constructed, and samples were tryptic digested and eluted using an Orbitrap Fusion Mass Spectrometer (LC/MS/MS) for proteomics analysis. The raw data were processed by Proteome Discoverer 2.2 and searched against the UniProt database using Sequest; the Scaffold toolkit quantified the identified proteins. Following quality control (e.g., principal component analysis) and data normalization, 9000 peptides corresponding to 1260 proteins were identified. Next, the differentially-expressed proteins were filtered through statistical analysis, including missing value imputation, data normalization, and t-test. The bulk of these proteins were within the range of 11–100 KDa; some were in the range of 100–200 KDa, and a few were in the range of 400–500 KDa. Overall, 187 differentially-expressed proteins (78 from the CM of CD36^−^ FBs and 109 from the CM of CD36^+^ FBs) had a p-value of less than 0.05 (Figure 7). Proteomic profiling was continued with additional bioinformatics analysis to identify ligands and exosomes and validate pertinent factors that induce growth suppression.

### 3.5. The Ligand-Receptor Bindings Are Identified

A total of 26 ligands, each with one or more receptors, were identified by compiling a master list from three databases of HPMR, STRING, and HPRD [35,36]. The ligand-receptor pairing in the CM of CD36^+^ FBs also indicated the enrichment of pathways involved in cytokine signaling of the immune systems, integrin cell surface interactions, insulin-like growth factor-2, and ECM organization. Table 2 shows the top four ligands that are overexpressed in the CM of CD36^+^ FBs, which were likely to induce growth suppression following bioinformatics analysis:

- TIMP2 has been shown to suppress the growth and metastasis of triple-negative breast cancer in a murine model by disrupting Wnt and PI3K signaling [37].
- Slit and Robo receptors are silenced in many types of cancer, play an important role in tumor growth, migration, and the tumor microenvironment [38], and have been suggested as a signaling pathway for tumor suppression [39].
- FBLN1, a glycoprotein member of ECM, is epigenetically downregulated in bladder cancer [40] and interacts with ADAMTS to reduce the proliferation of the breast cancer cell lines MCF7 and MDA-MB-231. ADAMTS-like protein 1 is also one of the differentially secreted proteins in the proteomics data.
- The literature on the PENK ligand in the context of growth suppression in breast cancer is minimal and very recent [41], but there is evidence that high expression of PENK is associated with favorable outcomes in patients with gastrointestinal stromal tumors [42].

Finally, 106 and 72 differentially-secreted proteins from the CM of CD36^+^ and CD36^−^ FBs, respectively, were included in the Vesiclepedia [43], which indicates that a large proportion of proteins are transferred through exosomes or microvesicles.

### 3.6. SLIT3, FBLN1, and PENK Induce Apoptosis in MDA-MB-231

**Figure 8:**
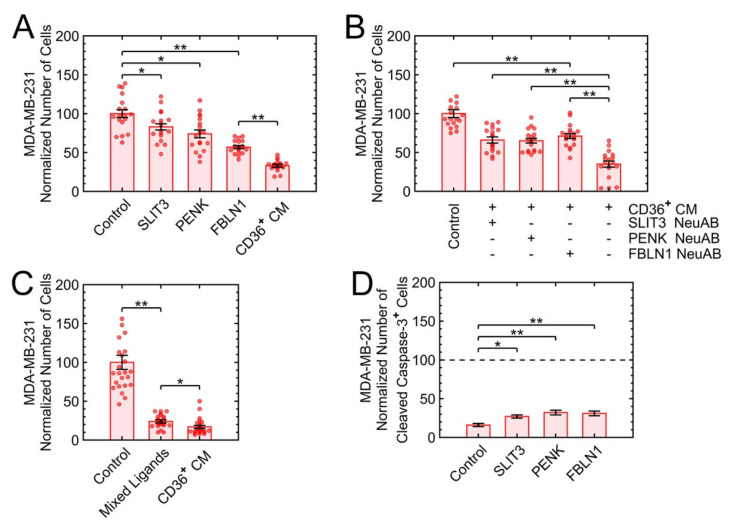
The mixture of three ligands added to the normal culture induces a similar level of growth suppression as the CM of CD36^+^ FBs. (**A**) The addition of each recombinant protein to the MDA-MB-231 medium induced partial growth suppression. (**B**) The addition of each neutralizing antibody to the CM of CD36^+^ FBs interfered partially with the growth suppression. (**C**) Mixed recombinant proteins of SLIT3, PENK, and FBLN1 induced an additive growth suppression that was similar to the CM of CD36^+^ FBs. (**D**) Protein ligands induced apoptosis via the caspase-3 pathway after one day of treatment. * *p* < 0.05, ** *p* < 0.001.

Candidate recombinant human proteins of Table 2 were commercially acquired. TIMP2 did not affect growth suppression (Figure S6A). The remaining three ligands were added one at a time and then as a mixture. Controls included the (i) neutralizing antibody for each of the corresponding recombinant proteins and (ii) colony formation assay for MCF10A. With respect to the concentration of recombinant proteins, we initiated with the published approximate ranges of SLIT3 [44] and FBLN1 [45,46] followed by serial dilution experiments of 0.1, 1, and 10 µg/mL for SLIT3 and FBLN1, and 0.1 and 1 µg/mL for PENK. SLIT3 and PENK at the concentration of 10 µg/mL and 1 µg/mL, respectively, induced growth suppression. However, FBLN1 induced growth suppression in a dose-dependent manner (Figure S6B–D). Each ligand, at its highest concentration, was added to the culture condition, and the growth suppression was quantified, with FBLN1 being the most effective (Figure 8A).

Additional controls included adding the neutralizing antibody of each ligand to the CM of CD36^+^ FBs separately, where the interference with the growth suppression was observed and quantified (Figure 8B). To investigate whether the three candidate ligands have an additive growth suppression effect, they were added at their highest concentrations to the 3D cultures of MDA-MB-231. The mixture indicated a similar growth suppression effect as the CM of CD36^+^ FBs (Figure 8C), and each ligand-induced apoptosis through the caspase-3 pathway after one day of treatment (Figure 8D). Finally, the toxicity of each ligand and their mixture were evaluated in premalignant 3D colonies of MCF10A with only FBLN1, at the high concentration, and mixture showing approximately 30% growth suppression in colony formation (Figure S7).

### 3.7. KLF10 Is One of the Transcription Factors Involved in Growth Suppression

**Figure 9:**
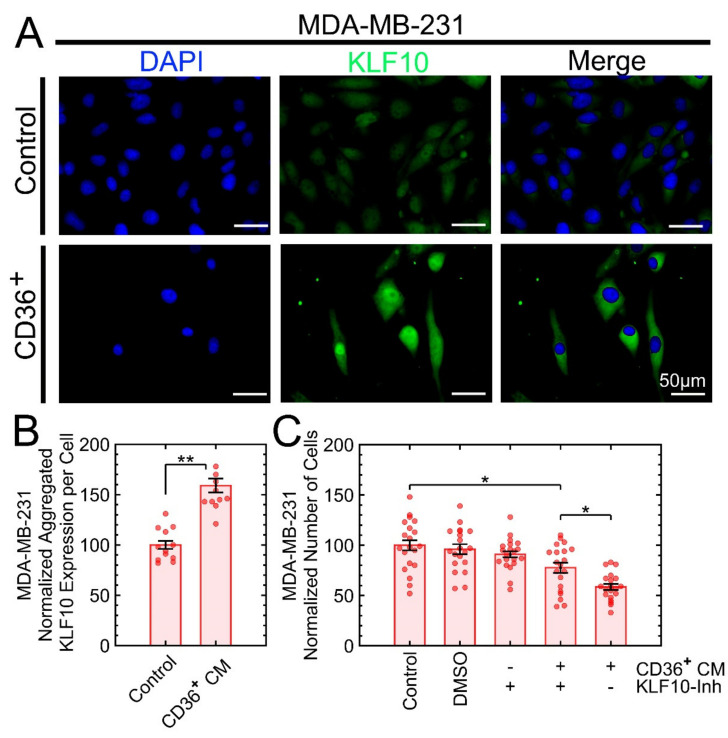
Overexpression of the KLF10 transcription factor is one of the mechanisms for growth suppression in MDA-MB-231 as a result of exposure to the CM of CD36^+^ FBs. (**A**) DAPI and KLF10 staining for control and treatment were monitored with fluorescent microscopy. (**B**) Quantitative analysis indicated overexpression of KLF10 with the treatment. (**C**) A control experiment to validate reduced growth suppression by adding the KLF10-inhibitor to the CM of CD36^+^ FBs. * *p* < 0.05, ** *p* < 0.001.

Model-based analysis of the regulation of gene expression (MARGE) [47] was used to predict the key transcription factors (TFs) corresponding to differentially expressed proteins in Figure 7. MARGE uses a comprehensive library of publicly available human H3K27ac ChIP-seq profiles and DNase I-hypersensitive regions to make inferences about the cis-regulation of gene expression by defining a regulatory potential for each gene summarizing nearby H3K27ac ChIP-seq signals. MARGE predicted the KLF family of TFs [48,49] with KLF15 (*p*-value < 1 × 10^−5^) as the top candidate and KLF10 and KLF11 that are structurally similar. KLFs play an important role in metastasis, including epithelial–mesenchymal transition (EMT), changes in ECM, invasion, and angiogenesis. For example, KLF15 has been suggested to be a novel tumor suppressor in breast cancer [50]. KLF10 and KLF11 are involved in TGFβ signaling and the regulation of Smad2/3 [51]. More importantly, KLF10 has been shown to have anti-proliferative effects and to induce apoptosis in various carcinoma cells [52]. Because we have already shown that the coculture of MDA-MB-231 with CD36^+^ FBs reverses the aberrant expression of pSmad2/3 in MDA-MB-231 [19], we opted to investigate KLF10, KLF11, and KLF15. At the protein expression level, only KLF10 was overexpressed in MDA-MB-231 when exposed to the CM of CD36^+^ FBs. It is feasible that low differential expression of KLF11 and KLF15 can be due to low transcription, instability of transcript, low translation, or protein instability and degradation. Additional control for this experiment was the KLF10 inhibitor, which interfered with the growth suppression when added to the CM of CD36^+^ FBs (Figure 9).

## 4. Discussion

Recombinant protein activin A downregulated the CD36 expression in normal FBs; however, once activin A was removed, the CD36 expression level increased. This was an extension of earlier results that increased production of activin A by tumor cells downregulating the CD36 expression in FBs, which induces the FBs to secrete more activin A and to initiate a positive feedback loop [19]. Since downregulation of CD36 is one of the CAF markers [9], it is plausible that the tumor microenvironment can be reprogrammed by factors secreted by the CD36^+^ FBs to induce tumor suppression and, in turn, decrease the production of activin A per our earlier findings [19]. These secreted factors were winnowed down to be protein ligands and proteins being transported by extracellular vesicles. The protein fractionation experiment revealed that proteins with different molecular weights were responsible for growth suppression in MDA-MB-231. In contrast, the exosome biogenesis experiments suggested that ligands played a more critical role in growth suppression than the proteins being carried by vesicles. Proteomic profiling of the CM of CD36^+^ and CD36^−^ FBs and further bioinformatics analysis predicted four ligands (TIMP2, SLIT3, FBLN1, and PENK) and, except for TIMP2, all induced growth suppression. Furthermore, the mixture of SLIT3, FBLN1, and PENK induced a similar level of growth suppression as the CM of CD36^+^ FBs. Finally, the KLF family of TFs was highly enriched as a result of MARGE analysis with KLF10, implicated in TGFβ signaling and the regulation of Smad2/3 [51], and validated to be overexpressed. However, multiple TFs must be involved in growth suppression because the inhibitor of KLF10 neither reverses growth suppression back to the level of control nor increases growth suppression to the level of the CM of CD36^+^ FBs (Figure 9). In addition, KLF10 must be overexpressed as a result of combined secreted ligands and vesicles since SLIT3 and FBLN1 marginally contribute to its overexpression (Figure S8). Furthermore, TFs must be cancer subtype-specific. For example, MARGE also predicted YY1, which is downregulated in MCF7 [19] and SKBR3 when exposed to the CM of CD36^+^ FBs. Downregulation of YY1 in MCF7 is associated with the reversal of aberrant basal and lateral polarity [19], but in SKBR3 proved to be ineffective, which is potentially due to its intrinsic mutations.

Secreted SLIT3, FBLN1, and PENK, by CD36^+^ FBs, appear to have a complementary role in growth suppression via the apoptotic pathway. SLIT3 is important in regulating the FBs activity and collagen synthesis in an autocrine manner, is involved in cell migration and tumor suppression [53], is shown to be low in lung tumor tissue [54]; however, treatment by SLIT3 inhibits the proliferation of malignant melanoma cells [38]. These observations suggest that the overall treatment by SLIT3 not only contributes to growth suppression and reduces secreted signaling factors (e.g., activin A) by cancer cells but also manipulates the FBs toward a more stable biological activity, thus potentially reprogramming the tumor microenvironment. FBLN1 is one of the regulators of TGFβ signaling [55], is downregulated in lung cancer [56], has been suggested to be a tumor suppressor by inducing apoptosis [40] and at the same time is antiapoptotic through Notch signaling [57], and its mechanism of action remains still unknown. In addition, FBLN1 was shown to be elevated in normal FB when compared to CAF [58]. High expression of PENK is associated with improved outcomes in GIST patients and is postulated to act as a tumor suppressor [42], inhibit breast tumor growth [41], and is required, in part, for induction of apoptosis through the NF-κB/p53 pathway [59]. Thus, it is plausible that SLIT3, FBLN1, and PENK cooperate and induce apoptosis through different pathways.

## 5. Conclusions

In this study, we extended our previous studies to show that the CM of CD36^+^ FBs induced growth suppression, via apoptosis, in a subset of breast cancer cell lines. We then profiled the CM of CD36^+^ versus CD36^−^ FBs to predict the active protein ligands of SLIT3, FBLN1, and PENK. Subsequently, for each active ligand, the range of concentrations responsible for growth suppression was identified. We then showed that a cocktail of SLIT3, FBLN1, and PENK, at their highest concentrations, can induce a similar amount of growth suppression as the CM of CD36^+^ FBs in MDA-MB-231.

Future studies will investigate both FBs and tumor cells. We will measure the exact concentration of each ligand and potential exosome in the secretome of FBs and examine how the secretome of FB is regulated through lipid uptake. We will also investigate the interactions of ligands and exosomes in growth suppression, restoration of the normal functioning of CAFs through an autocrine loop, and recruitment of other stromal cells. The latter is evident by the loss of CD36 in macrophages and endothelial cells, leading to inflammation [60] and neoangiogenesis [61], respectively. These observations are complemented by the role of (a) SLIT3 in promoting monocyte migration [44], and (b) FBLN1 in tumor immunosurveillance, and improved survival in the presence of lymphoid infiltrates [61]. In summary, there is overwhelming evidence that these ligands can also contribute to novel therapies in cancer.

## Figures and Tables

**Table 1:** Details of the immunofluorescent staining for each molecular endpoint.

Target	CD36	YY1	Ki67	KLF10	Cleaved Caspase-3	Cyclin E1
Permeabilization (Triton X-100)	0.5%	0.5%	0.5%	0.5%	0.3%	0.5%
Blocking Solution (BSA)	1%	1%	1%	1%	5% BSA + 0.3% Triton X-100	1%
Primary Antibody	Santa Cruz SC-7309 Or Novus Biological NB 400-144	Abcam Ab232573	Abcam Ab16667	Novus Biological NBP3-04586	Abcam Ab32042	Novus Biological NBP2-67760
	1:250	1:250	1:500	1:100	1:100	1:100
Secondary Antibody	Abcam Ab150113 Or Ab150077	Abcam Ab175471	Abcam Ab150077	Abcam Ab150077	Abcam Ab150077	Abcam Ab150077
	1:250	1:250	1:500	1:250	1:250	1:250

**Table 2:** Four potential candidate ligands (Figure 7) in the CM of CD36^+^ FBs are listed.

Ligand	Receptors	*p*-Value	Log2 (Fold Change)
TIMP2 (24 KDa)	ITGA3, ITGB1	0.00005	0.7
FBLN1 (77 KDa)	ITGB1	0.001	0.5
PENK (31 KDa)	MRGPRX1,	0.001	0.5
	OGFR,		
	OPRD1,		
	OPK1,		
	OPRM1		
SLIT3 (168 KDa)	ROBO2	0.03	0.7

## Data Availability

The data presented in this study are available in this article (and Supplementary Materials).

## Supplementary Materials

The following are available online at https://www.mdpi.com/article/10.3390/cancers13184521/s1, Figure S1: CD36 is overexpressed in fibroblasts (FBs) of the normal mammary gland. The image was collected from the Human Protein Atlas, Figure S2: CD36 expression in fibroblasts (FBs) reverses once activin A is removed, Figure S3: The CM of CD36^+^ FBs has no impact on the size of colonies for SKBR3 cell line event though the frequency of high-expressing YY1 colonies is decreased, Figure S4: Both acute (2 days) and continuous (7 days) exposures induce growth inhibition, with continuous exposure having a higher growth suppression, Figure S5: There is no growth arrest in G1/S boundary, in MDA-MB-231, as a result of exposure to the CM of CD36^+^ FBs, Figure S6: Serial dilutions of each of the four recombinant proteins indicate that only SLIT3, FBLN1, and PENK induce growth suppression, Figure S7: At the highest concentration of recombinant proteins, only FBLN1 and mixed ligands inhibited colony formation for MCF10A, Figure S8: SLIT3 and FBLN1 contribute marginally to the overexpression of KLF10 in the 3D culture of MDA-MB-231.
