# Supplementary material for: Protein Ligands in the Secretome of CD36+ Fibroblasts Induce Growth Suppression in a Subset of Breast Cancer Cell Lines
Source: Cancers (Basel). 2021 Sep 8;13(18):4521. doi: 10.3390/cancers13184521 (PMC8469330; doi:10.3390/cancers13184521)
Supplement: Supplementary file 1 [file cancers-13-04521-s001.zip › cancers-1358419-supplementary.pdf]

Supplementary Material: Protein ligands in the secretome of CD36⁺ fibroblasts induce growth suppression in a subset of breast cancer cell lines

Kosar Jabbari, Garrett Winklemaier, Cody Andersen[1], Paul Yaswen, David Quilici, Saori Furtua, Qingsu Cheng, and Bahram Parvin

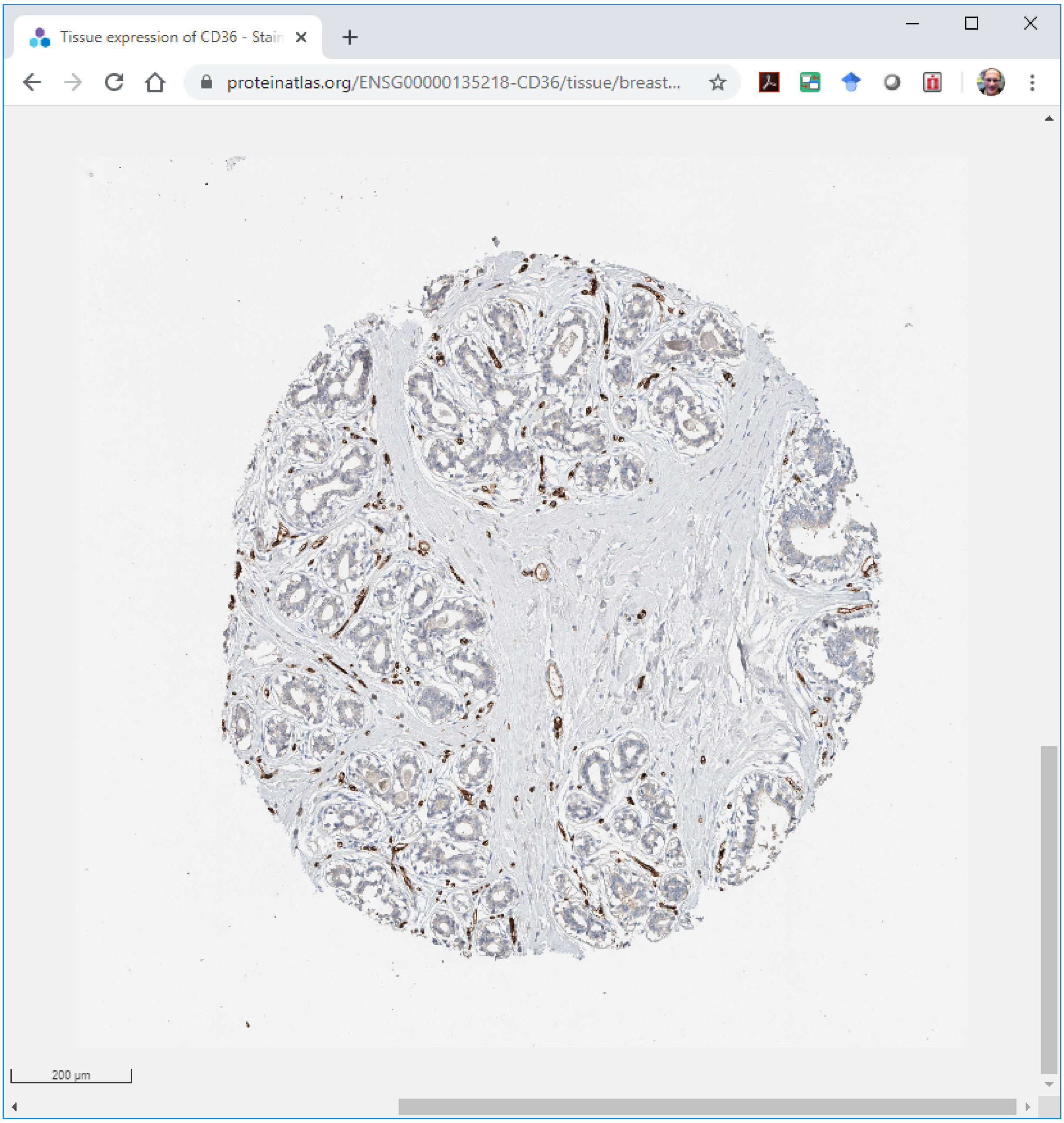

Figure S1: CD36 is overexpressed in fibroblasts (FBs) of the normal mammary gland. Image is queried from the Human Protein Atlas.

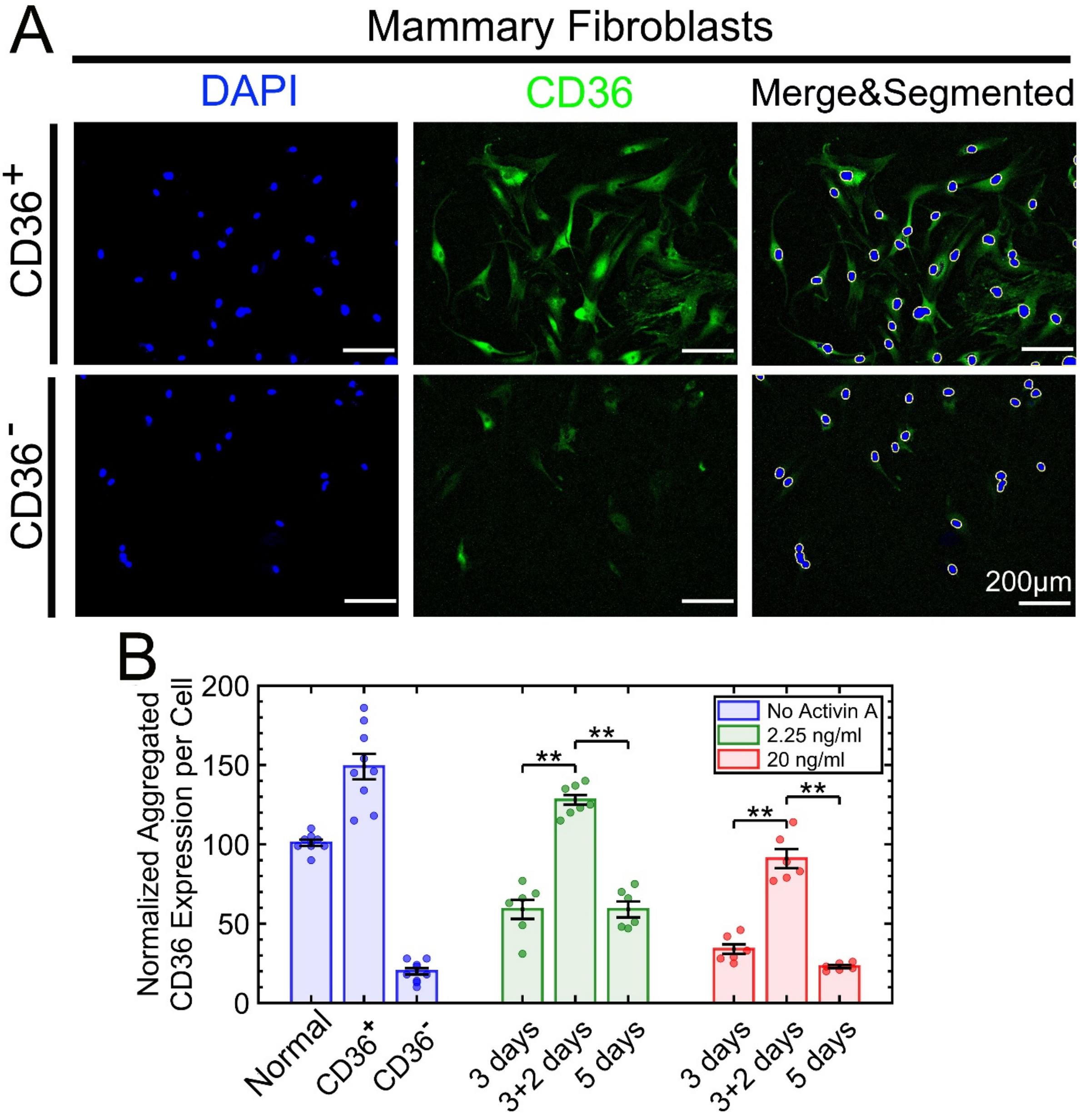

Figure S2: CD36 expression in fibroblasts (FBs) reverses once activin A is removed. (A) Nuclear counterstain (DAPI) with the expression levels of $CD36^{+}$ or $CD36^{-}$, in the FITC channel, monitored by fluorescent microscopy. Segmented DAPI is overlaid on the FITC channel (last column). (B) The blue bar corresponds to the untreated FBs. Green and Red bars correspond to the normal primary FBs exposed to nominal (2.25 ng/ml) and high (20ng/ml) concentrations of activin A, respectively. For all conditions, the CD36 expression was normalized to the untreated normal primary FBs. **$p<0.001$.

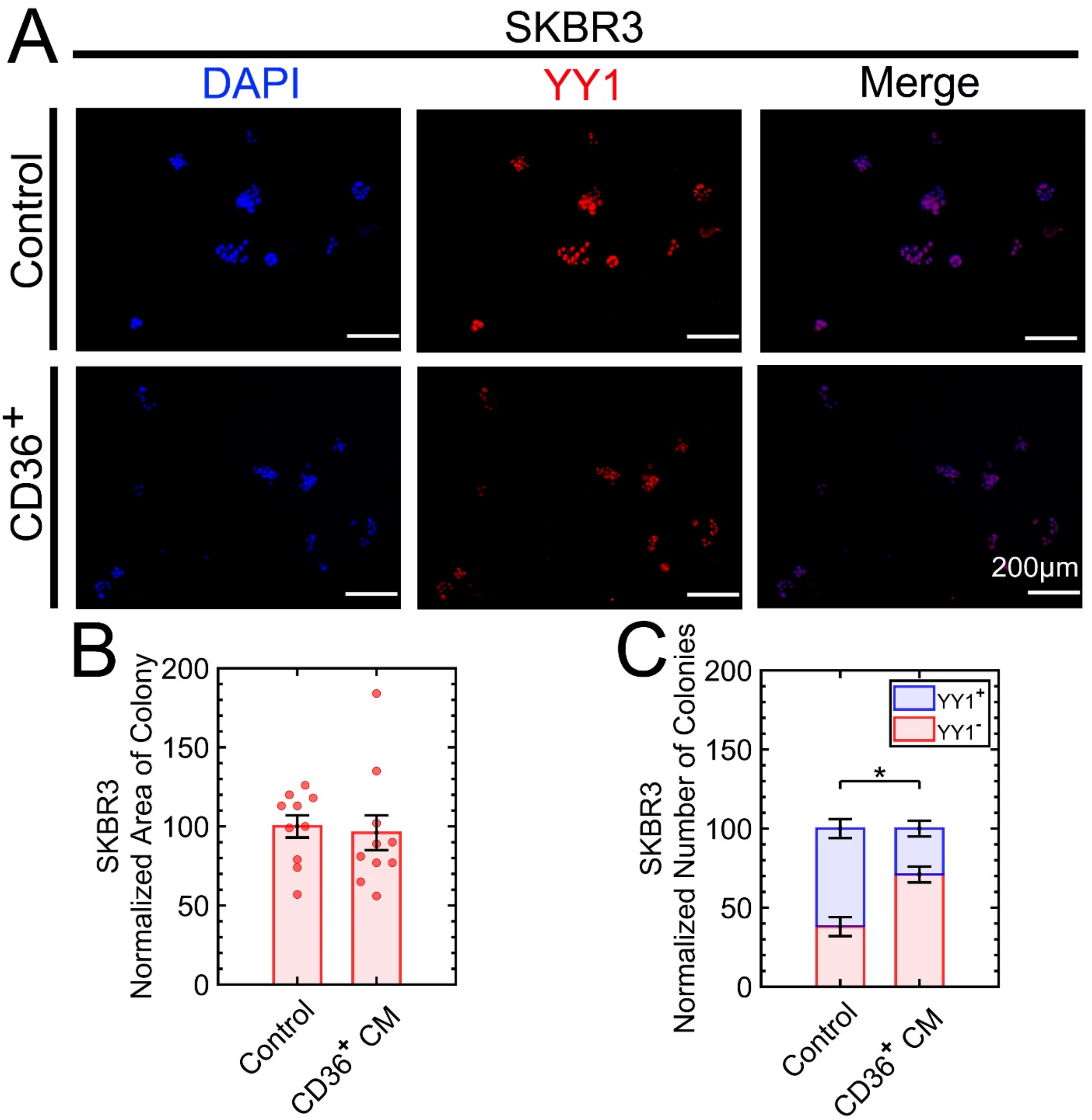

Figure S3: The CM of CD36+ FBs has no impact on the size of colonies for SKBR3 cell line event though the frequency of high-expressing YY1 colonies is decreased. (A) SKBR3 colonies are stained with DAPI and YY1 and visualized by fluorescent microscopy. (B) Quantitative analysis of the size of the colonies indicated no difference between control and treatment. (C) Two populations of YY1-expressing colonies were present, with the frequency of high-expressing YY1 levels being reduced as a result of exposure to the CM of CD36+ FBs. *$p<0.05$

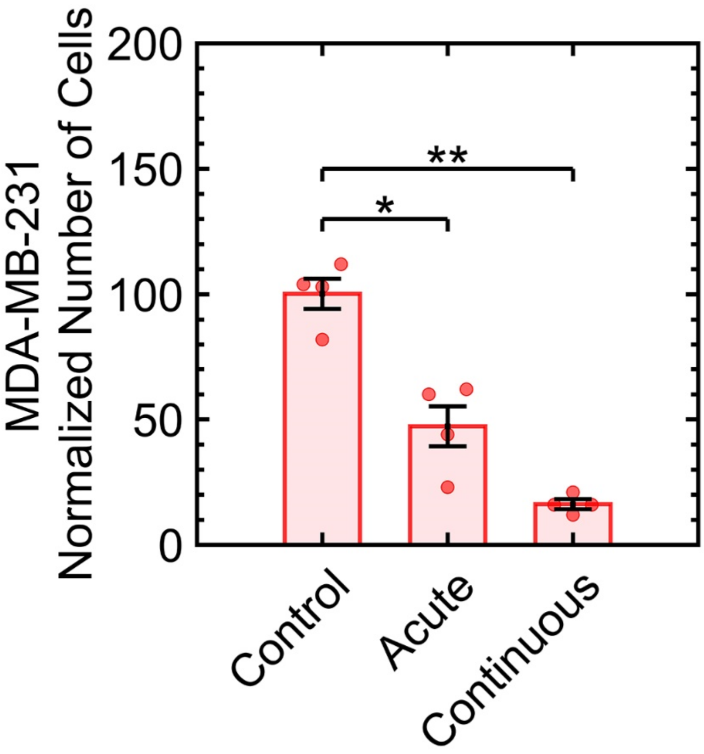

Figure S4: Both acute (2 days) and continuous (7 days) exposures induce growth inhibition, with continuous exposure having a higher growth suppression. *p<0.05, **p<0.001.

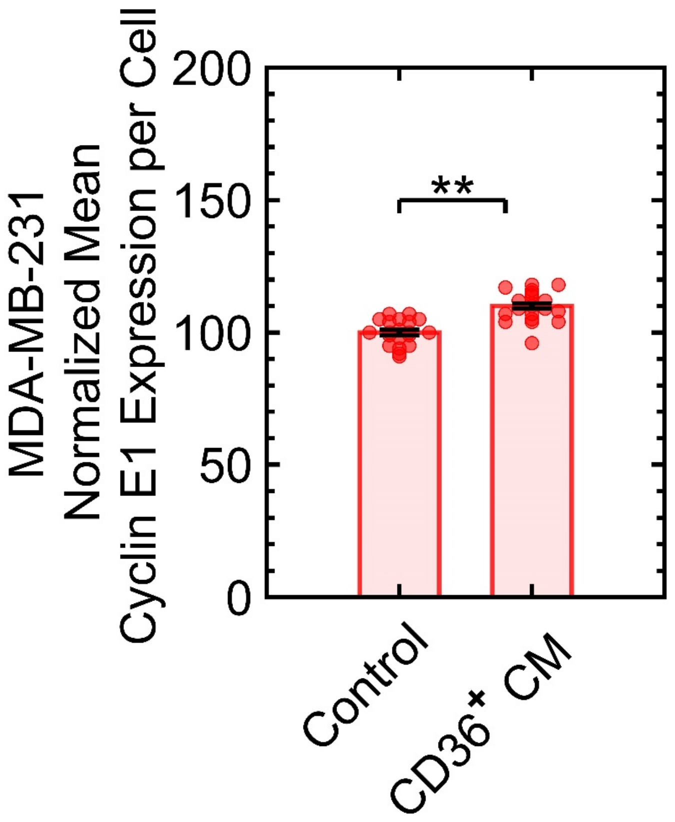

Figure S5: There is no growth arrest in G1/S boundary, in MDA-MB-231, as a result of exposure to the CM of CD36+ FBs. *p<0.05, **p<0.001.

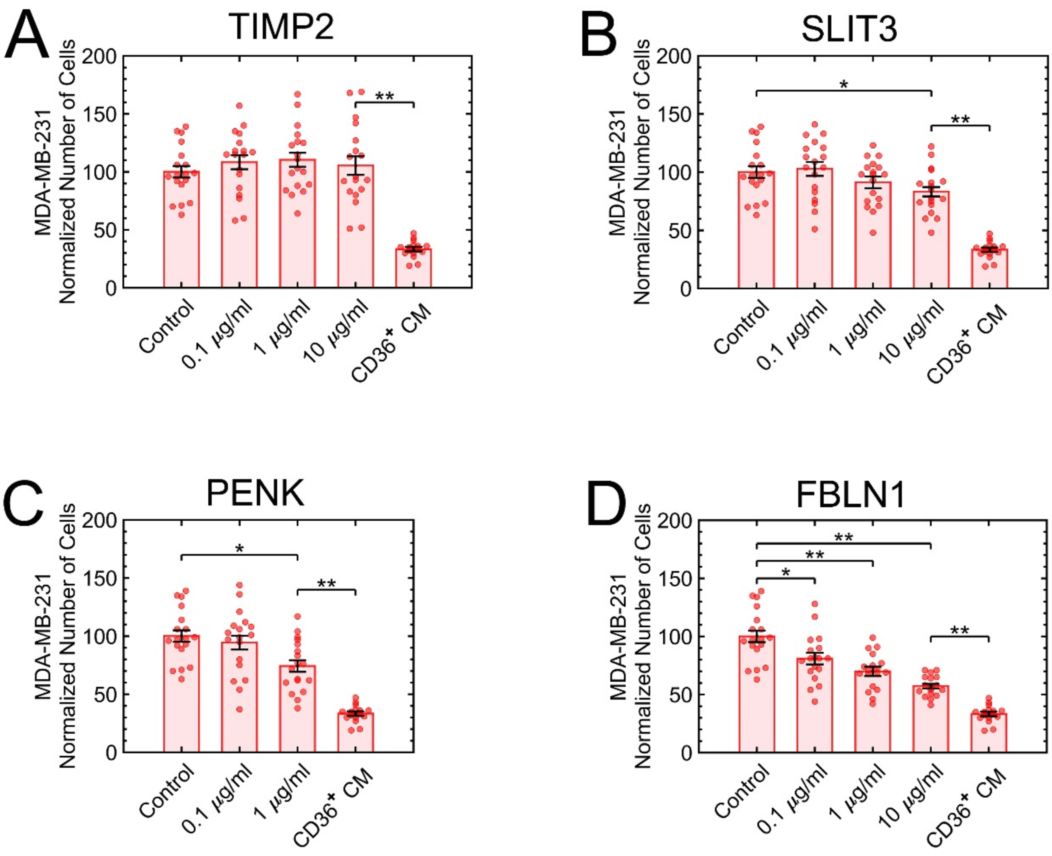

Figure S6: Serial dilutions of each of the four recombinant proteins indicate that only SLIT3, FBLN1, and PENK induce growth suppression. (A) TIMP2 induced no growth suppression. (B) SLIT3 induced growth suppression at 10μg/ml. (C) PENK induced growth suppression at 1μg/ml. (D) FBLN1 induced growth suppression at 0.1μg/ml. *$p<0.05$, **$p<0.001$.

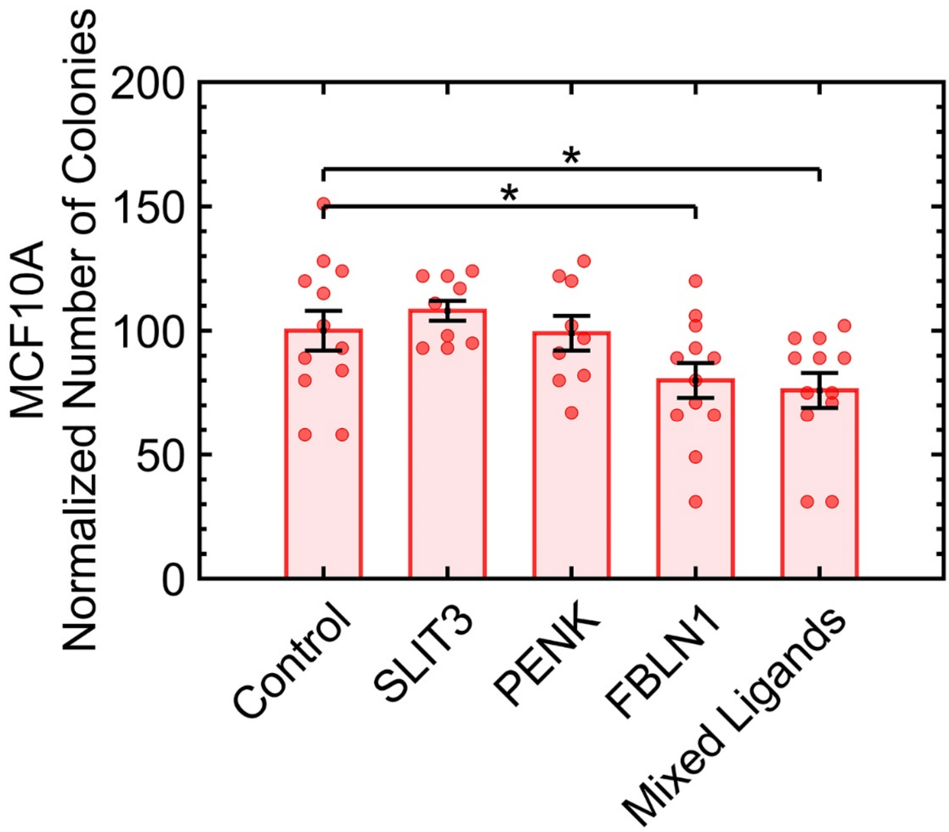

Figure S7: At the highest concentration of recombinant proteins, only FBLN1 and mixed ligands inhibited colony formation for MCF10A.

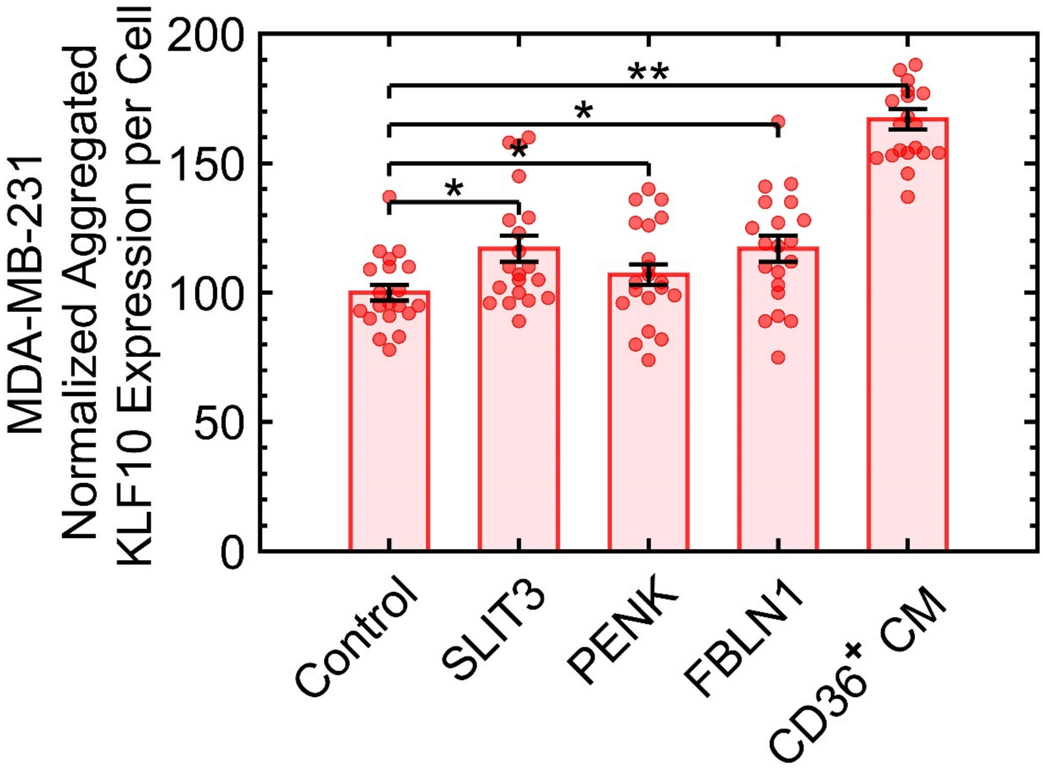

Figure S8: SLIT3 and FBLN1 contribute marginally to the overexpression of KLF10 in the 3D culture of MDA-MB-231
